# Exploring the Dynamics of Attracting and Retaining Acute Care Psychiatric Registered Nurses: An In-Depth Analysis Using Focus Groups

**DOI:** 10.1155/2024/3167255

**Published:** 2024-04-09

**Authors:** Kendra L. Ratnapradipa, Julia F. Houfek, Phoebe Gearhart, Erin O. Schneider, Christine Chasek, Danae Dinkel, Katrina M. Cordts, Marley Doyle, Priyanka Chaudhary, Deepanjali Bhale, Shinobu Watanabe-Galloway

**Affiliations:** ^1^Behavioral Health Education Center of Nebraska (BHECN), University of Nebraska Medical Center, 984242 NE Medical Center, Omaha, NE 68198-4242, USA; ^2^Department of Epidemiology, College of Public Health, University of Nebraska Medical Center, 984395 Nebraska Medical Center, Omaha, NE 68198-4395, USA; ^3^College of Nursing, University of Nebraska Medical Center, 985470 Nebraska Medical Center, Omaha, NE 68198-5470, USA; ^4^Department of Psychiatry, Nebraska Medicine, Omaha, NE 68198-5578, USA; ^5^Department of Counseling, College of Education, Health & Human Sciences, University of Nebraska Omaha, Omaha, NE 68182, USA; ^6^School of Health and Kinesiology, University of Nebraska Omaha, Omaha, NE 68182, USA; ^7^Department of Neurological Sciences, College of Medicine, University of Nebraska Medical Center, 985920 Nebraska Medical Center, Omaha, NE 68198-5920, USA; ^8^Department of Psychiatry, College of Medicine, University of Nebraska Medical Center, 985575 Nebraska Medical Center, Omaha, NE 68198-5575, USA

## Abstract

The recruitment and retention of in-patient psychiatric mental health registered nurses (PMH-RNs) remains a challenge. This qualitative study sought to identify factors impacting the recruitment and retention of PMH-RNs in acute-care settings. Participants (*N* = 15) were recruited for focus groups including one with in-patient unit administrators (*n* = 4), two with current PMH-RNs (*n* = 7), and two with nursing students (*n* = 4). Data were analyzed using a directed content analysis approach. Participants were informed about the study's purpose, procedures, potential risks, and benefits, and they provided verbal consent before participating. Administrators emphasized a focus on retention and described a variety of supports they provided PMH-RNs, including formal and informal support and education. PMH-RNs' most prevalent concerns were their safety, co-worker and/or management challenges, and emotional and/or physical exhaustion. Students also expressed concerns about safety in psychiatric settings and desired more interaction with PMH-RNs. They were also interested in learning more about the specialty as they valued the opportunity to see change in patients. All three groups mentioned a need for more interaction between students and PMH-RNs, while safety concerns were expressed by both PMH-RNs and students. Because PMH-RNs play a critical role for in-patient psychiatric care, recruiting and retaining specialist nurses can focus on increasing student engagement with the PMH-RNs, attending to PMH-RNs' management and staffing concerns, and providing additional resources for responding to events that threaten safety in the workplace to prevent burnout.

## 1. Introduction

The US has experienced an increased demand for mental health services, in part due to sequelae from the COVID-19 pandemic [1, 2]. A 2022 national survey found that while 42% of US adults reported needing mental health care and 24% reported needing substance use care in the previous 12 months, 43% of respondents also indicated they did not receive needed care [3]. Barriers to accessing mental health care include shortages of mental health providers, geographic maldistribution of providers, a decrease in psychiatric in-patient beds, and a lack of knowledge about how to access care [3–6].

The increased need for mental health care in light of the pandemic has also affected clinician well-being, prompting a call to recognize and address provider stressors [7]. Workplace violence in psychiatric settings, including abusive incidents and threats to safety, affects care delivery and provider health [8, 9]. Psychiatric mental health nursing (PMHN) has responded to the ongoing shortage of mental health providers and these workplace stressors by defining and characterizing the PMHN workforce, describing strategies to meet provider shortages, and encouraging PMHN to champion clinician well-being [5, 10–16].

The PMHN workforce, comprised of both Registered Nurses (RNs) and Advanced Practice Registered Nurses (APRNs), is the second largest group of US mental health professionals [13]. PMHNs play a unique and critical role in providing interdisciplinary, collaborative mental health treatment and care [11, 13]. The majority of PMH-RNs reported practicing in a hospital setting [12]. PMH-RN responsibilities include assessing mental health, applying the nursing process to develop diagnoses and care plans, and administering and monitoring psychobiological treatments [17].

Recruitment and retention of PMHN has been a long-standing concern for the specialty [18–21]. A 2022 survey by Nursing Solutions, Inc. (NSI) of 273 US hospitals reported the turnover rate for PMH-RNs was 20.8%, slightly lower than the 2021 rate of 23.4% and lower than the 2022 average turnover for RNs (22.5%) employed by reporting hospitals [22]. However, the NSI study also reported that behavioral health units experience a 100% cumulative turnover rate of PMH-RNs over five years [22]. Responding hospitals reported that the top ten reasons (in descending order) for RNs leaving their positions were: personal concerns, career advancement, relocation, salary, retirement, unknown reasons, education, scheduling, workload or staffing ratios, and commute. Three of these reasons (retirement, education, and career advancement) are also supported in the literature about psychiatric nursing [11, 12, 23]. A recent PMHN workforce survey found that over half (52.6%) of PMH-RN respondents were in their 50s and 60s, indicating an aging workforce nearing retirement [12]. This 2022 workforce survey found that the number of PMHNs completing a graduate degree since 2010 almost doubled [23], suggesting that at least a portion of PMH-RNs pursue a graduate degree to advance their nursing careers. A reason that may underlie other turnover factors cited in the NSI study is the need for support and mentoring of RNs new to PMHN practice [19], such as nurse residency programs [21], and continuing education focusing on best PMHN practices [18]. Finally, due to stigma, psychiatric nursing is not seen as a desirable or valuable nursing specialty and may hinder recruitment of students and RNs into PMHN [11, 19, 23].

Recruitment and retention of PMH-RNs are crucial in mental healthcare shortage areas. It is estimated that the shortage of mental health providers across the US affects 163 million people who need care [24]. Nebraska is one state that has a severe shortage of mental health providers, with 90 of 93 counties designated as mental healthcare shortage areas. While an estimated 3.7% of US RNs cited PMH/substance abuse as their primary nursing specialty [25], only 2.9% of Nebraska RNs and APRNs cited PMHN as their primary practice during license renewal [26]. According to a 2020 survey, 10% of urban and 44% of rural responding Nebraska healthcare facilities that employed PMHN contracted for these nurses in the past six months citing lack of applicants, lack of qualified nurses to fill positions, and a need to cover for the absence of regular staff [27]. Two-thirds of the urban healthcare facilities and half of rural facilities who responded to a question about time needed to fill psychiatric nursing positions indicated recruitment spanned four or more months.

Because PMH-RNs' provide essential care, it is important that we better understand PMH-RNs choice of speciality and working environments that promote or deter their recruitments and retention. The Behavioral Health Education Center of Nebraska (BHECN) is a state-funded behavioral health workforce development center with a mission to recruit, train, and retain the behavioral health workforce. To accomplish its mission, BHECN has an interest in understanding PMH-RNs' perspectives related to choosing to practice psychiatric nursing and conditions which help or hinder providing psychiatric nursing care. Therefore, we conducted a study to describe: (1) reasons for PMH-RNs staying or leaving their acute-care psychiatric nursing position and (2) recommendations to improve recruitment, retention, and training of PMH-RNs. Nurse managers are directly responsible for the recruitment and retention of nursing staff and for safe, effective working environments. Results of this study will provide knowledge of PMH-RNs' perceptions of their work environments and their role in clinical care. Understanding these perspectives can help nurse managers provide responsive clinical leadership to help maintain and grow the PMHN workforce.

## 2. Conceptual Background

Behavioral health workforce development is guided by a Career Pathway or “pipeline” approach for recruitment and retention of providers, including psychiatric mental health nurses [28, 29]. This heuristic provides a systematic approach to career development starting with career education in elementary school and continuing through completion of postsecondary coursework and entry into the workforce [30]. The Pathway approach includes formal or informal mentoring of students by practicing providers. It also includes students' exposure to actual practice environments through clinical experiences as part of their academic coursework, internships, and observational experiences. Encouraging and mentoring interested students to explore behavioral health careers can stimulate and sustain their interest in these careers [31]. Interacting with the Career Pathways approach are academic-practice partnerships in nursing. These partnerships aim to strengthen collaboration between schools of nursing and health care institutions to improve the quality of both nursing education and clinical nursing care [32]. Academic-practice partnerships can provide the infrastructure for mentoring aspiring psychiatric mental health nurses as well as foster nurses' continuing career development to promote retention. To assure that recruitment and retention efforts for psychiatric mental health nurses address local needs [33], we used qualitative methods to explore PMH-RNs reasons for staying or leaving their acute-care psychiatric nursing positions and their related recommendations for recruitment and retention of PMH-RNs.

## 3. Materials and Methods

Prior to data collection, the study was screened by the University of Nebraska Medical Center's Institutional Review Board. Because BHECN is a workforce development center and the study was asking about professional practice, the study did not constitute human subjects research as defined at 45CFR46.102 and was exempt from IRB oversight. The study was conducted in accordance with ethical principles including (verbal) informed consent prior to beginning the audio recording. Transcripts were blinded to preserve participant anonymity.

### 3.1. Participants

Inclusion criteria included being from Nebraska institutions or facilities and being an administrator who supervised PMH-RNs in acute-care settings (*n* = 4), a nurse who practiced as a PMH-RN with current or prior experience in acute-care settings (*n* = 7), or a nursing student who had a psychiatric rotation (*n* = 4).

### 3.2. Recruitment

We used targeted recruitment through partnerships and personal contacts to advertise the study to organizations and institutions that employ PMH-RN. Additionally, BHECN collaborates with a network of academic programs whose mission is to develop the behavioral health workforce, including the three psychiatric-mental health graduate nursing programs in the state, which advertised the study to students.

### 3.3. Focus Group Facilitation Guides

Separate facilitation guides were developed following a literature review to identify potential reasons for PMH-RNs leaving their jobs: (1) lack of training to gain necessary skills and knowledge to fulfill PMH-RN responsibilities [18, 34, 35], (2) bullying by other PMH-RNs [36], (3) workplace violence [8, 9], (4) lack of administrative support [37], and (5) lack of resources to improve mental health of PMH-RNs [38].

### 3.4. Data Collection

Five groups (one administrator, two PMH-RN, and two student groups) were held to accommodate scheduling. Sessions lasted approximately 60 minutes and were facilitated by a PMHN, a social worker, and an epidemiologist between December 14, 2021 and March 18, 2022. The PMHN was prepared to address potential participant distress in discussing workplace violence. Focus groups were conducted via Zoom, audio recorded, and transcribed verbatim. Participants received a $50 gift card for their participation.

### 3.5. Data Analysis

Interviews were uploaded into NVivo for analysis using a directed content analysis approach [39, 40]. Two researchers with extensive qualitative analysis experience read through the transcripts several times. The lead analyst (DD) developed codes and a codebook identifying main categories through a deductive approach, which followed the interview guide and inductive development of subcategories along with definitions for each category of participants. A second researcher (PC) reviewed all coding, noting discrepancies, and the two met to resolve conflicts and reach consensus.

The analysts presented the initial coding structure to the research team who provided feedback. Next, the lead analyst reviewed and revised the data based on the feedback, making minor revisions to the codebook. The second analyst then reviewed all coding a final time, noting discrepancies and the two researchers met again to discuss until reaching consensus. Anchor quotations were identified for each main theme. Data credibility relied on data triangulation from the perspectives of administrators, nurses, and students and investigator triangulation by obtaining coding feedback from multiple researchers [41]. An audit trail was used for dependability and confirmability [41].

## 4. Results

Fifteen people participated in five focus groups: one administrator group (*n* = 4), two PMH-RN groups (*n* = 4, *n* = 3), and two nursing student groups (*n* = 2, *n* = 2). All administrators and students were females. Two PMH-RNs were male and the remaining were females. All PMH-RNs had left acute-care and were working in outpatient settings at the time of the study. They had 71 years of cumulative experience (range 1–37 years) in psychiatric nursing in any setting, but primarily in acute-care units.  Table 1 summarizes the themes by participant category with illustrative quotations.

### 4.1. Administrators

#### 4.1.1. Recruitment Challenges Lead to a Focus on Retention

Recruitment was a significant challenge identified by administrators. The pandemic was mentioned as a contributing factor, citing nurses' ability to obtain higher wages at other health care organizations and the challenges of the pandemic increased older nurses' desire to retire. An administrator said, “We had five agency nurses that were on two back-to-back contracts, and they all left at the same time when COVID[-19] kind of changed their income and so they took contracts elsewhere.” Other known reasons for nurses leaving were a desire to work with a different population or to have more flexible hours. Despite challenges, administrators felt positive that recruitment was improving as they started to get more candidates and were filling open positions. An administrator described how six to eight months prior they only had a few candidates for open positions but now “we've had three offers out waiting on two, one has accepted so we are now seeing folks coming and wanting to come into behavioral health.”

Employee retention was a major focus, viewed as a long-term process that starts with recruitment and “pipeline” development. One strategy was precepting students as part of concentrated clinical learning experiences. A participant noted, “We'll be precepting two students that have an interest in Psych…and so we would be interested in offering them positions if things work out and so (we are) kind of excited to have that opportunity.” Another noted the importance of starting early and a need “to keep working on getting started in high schools and getting kids interested in mental health.”

Administrators also indicated their organizations were increasing wages and/or providing support to reduce stress to improve retention. As one participant mentioned, “We have another process that if a staff is injured, we follow up with them and several people involved in that and really doing a dive into supporting them through that process, and I think all of that has been helpful for retention.” While much of the discussion revolved around trying to recruit and retain nurses in the short term, one administrator did note the importance of other programming to ensure they stay long term.

#### 4.1.2. Ongoing Processes and Support for Nurses

Administrators had a variety of processes to onboard new nurses to their facility, including a mixture of online and in-person training with mentorship from more experienced nurses. Administrators appeared to primarily focus on individualized onboarding, which was often based on the nurses' previous experience as well as the type of position (e.g., contract vs. full-time).

Administrators mentioned a wide offering of support for their staff, including formal processes (e.g., Employee Assistance Programs (EAP), wellness committee events), informal processes (e.g., mentor), and education or training (e.g., ways to prevent lateral violence or hostility between co-workers). While none of the administrators discussed any instances of workplace bullying or violence, two mentioned they had direct training about it. For example, one administrator said, “So we have a policy on workplace violence, and we put a lot of effort around staff doing incident reports, if they're witnessing it or if it's happening and to come forward, there's an anonymous way to report it.”

Specifically when discussing how they supported staff following a violent event or altercation involving a patient, administrators mentioned they primarily focused on convening huddles immediately following the event to debrief. If administrators noticed a staff person needing additional support following the event, they would individually reach out. As one participant noted, “The debriefing and then if it's very apparent that somebody really needs some support, then we individually reach out to them and just offer that assurance and refer them to EAP if need be.”

#### 4.1.3. Needed Support for Nurses and the Profession

When discussing ways to better support PMH-RNs and the nursing profession in general, administrators identified several ideas including a need for care options, in-depth or triage support, and engaging high school students. Regarding a need for more care options, two administrators mentioned the lack of facilities for extremely violent patients as a challenge. An administrator explained, “you're often looking at months (to get them into a more secure facility); we are not equipped to handle people that are extremely violent to other staff…as well as to other patients.” Another administrator described a need for better triage support following staff injury. This administrator stated “…we brought in individuals who were trained in crisis intervention, how to deal with a violent episode to kind of work through those feeling…I think the ability to have a 911 (emergency) support person to come in and triage, that would be great.”

### 4.2. Nurses

#### 4.2.1. Experience and Challenges of Their Profession

All PMH-RNs described how they valued their profession and found it rewarding. One nurse said, “It's just very rewarding watching people recover from a very, very dark place and just seeing that personal growth and self-discovery.” However, all the nurses had left acute care and approximately half had considered leaving PMHN altogether. Despite a variety of challenges expressed, the most often expressed concern was about their own safety and previous encounters with violence. As one participant explained, “I've worked in some places that have been really unsafe where staff are getting seriously injured or patients are getting injured…and I'm not willing to work in a unit or a facility that has those core safety concerns.” Other top concerns were challenges with co-workers or management and feeling unappreciated. One nurse described a previous facility and why she left, “…the lateral incivility at work, leadership didn't listen at all, and there was no training there.”

Nurses expressed they were emotionally and/or physically exhausted and that the COVID-19 pandemic led to burnout. As one nurse noted, “…burnout (has been) a huge aspect (with) psych nurses just because our patient care is so difficult…but COVID[-19] especially made it worse.” Mask policies during the COVID-19 pandemic also presented a challenge. “When you have someone who's in psychosis or manic and very paranoid, having to wear a mask around them hinders your ability to form any kind of therapeutic relationship.” Other concerns or challenges mentioned by nurses included dealing with staffing shortages, a lack of community support or understanding for mental health, and maintaining work/life balance. For example, one nurse stated, “I think that a lot of times people, when you tell them you work in Psych, they have an idea of what that means and it is not what it really means…I don't think people understand mental illness at all.” The need to provide staffing seven days a week for in-patient settings was also a challenge. One nurse mentioned, “It's hard for my family, I have a baby at home and it's hard to work every other weekend and to be obligated to work holidays as he's growing up, I'm not sure I'm willing to do that.”

#### 4.2.2. Sources of Current Support

Like the administrators, nurses described various formal (e.g., EAP and wellness committee events) supports, although not all participants were familiar with these resources. The primary source of informal support focused on conversing with peers and veteran staff. For example, one nurse said, “That's where I learned most of my stuff is from veteran nurses who have been through it, and so venting to them after a situation like this (a violent event or situation) really is helpful to me and to get their support and feedback about what happens.” Nurses also discussed various self-care strategies that supported their mental and physical well-being, including conversing or spending time with friends, exercising, spending time alone or with family, although some of these practices were impacted by the pandemic. One nurse said, “Exercise for me, but you couldn't go to the gym with COVID[-19].” Consistent with the administrators, nurses mentioned the primary support following a violent event was through debriefing huddles. One nurse stated, “Everybody involved in the situation will always get together and we kind of talk about what happened, what went well, what didn't go well.”

#### 4.2.3. Need for Additional Support

Although the PMH-RNs mentioned huddles as a supportive process, not all the participants felt they were useful as implemented. A participant explained how the huddles actually led to increased stress by taking involved staff away from their other duties, particularly when working on an under-staffed unit.

*“It was just kind of, OK, we have to do this*…*We're going to have, you know, 30 seconds and we're just going to move on, without really the full weight of what can we learn from this situation*…*In spite of conversations that were had, (administrators) were very good about superficial level saying, yeah, we were listening, we care, but we're not going to do anything different than this process*…*”*

*“Or we have this safety huddle, which most of the people that seem to go to that seemed to have all the time in the world to spend all this time discussing all this stuff. I was like, I have to get back down there, there's only one person down there. The environment, it just didn't seem like the rest of the world understood what was actually happening on the unit and how really unsafe at times*…*”*

PMH-RNs described multiple needs for additional support for themselves and the profession. One frequently mentioned need was for additional staff at in-patient facilities; however, this was usually in reference to prior positions. Another requested item was more support following a violent event, exemplified by “there needs to be…more support for that because it can be physically as well as mentally damaging to be assaulted.” Relatedly and like administrators, PMH-RNs mentioned a need for more care options, including residential options and housing. Other support for current PMH-RNs included more education and more support from co-workers. One nurse stated, “We need to find courage from each other. So, if we just had a little more support, I believe the emotional stuff we go through would be beneficial.”

To support interest in their profession, another top need identified was for increased interaction with students. PMH-RNs had different ideas on how to best do this, such as having a mandatory psychiatric nursing clinical rotation or having opportunities to present to students to explain the psychiatric nursing role. One nurse described that in her first job, she would “… talk about what I did and what my career was, I think that if real psych nurses did that in schools, it could be beneficial.”

### 4.3. Students

#### 4.3.1. Mental Health Experience

Students' clinical rotation experience varied in length (8–14 weeks) and the number of sites (1–3). When discussing their experiences with mental health patients, students most often described their experience through either their own work, personal experience, or other clinical rotations. A student said she had been “a CNA (certified nursing assistant) for the last 10 years…we do see a lot of psych cases…so that is kind of helpful to have that experience.” Regarding experiences in other rotations, a student noted, “I feel like we've gotten to see a good variety of psychiatric diseases and mental health disorders and all of that just on like Med Surg floors and everything.” However, students felt like they had gained little experience with PMH-RNs and had a limited understanding of what PMH-RNs actually do. One student stated, “A lot of what they do is passing meds and making sure everyone is compliant with their medication.” While another student said, “I think a lot of students don't really understand it.”

#### 4.3.2. Concerns about the PMH Specialty

Students' primary concern was safety and experiencing violent patients. A student mentioned concerns she heard from other students, “I've been hearing it…just (students) thinking it's all violent patients but it's not. I think that's a big thing that worries some people.” Another stated “I think for me, at least, a big concern with that is safety. A lot of what I've seen is nurses not handling the situation as well as they could of.” The other major concern about PMHN was the stigma associated with mental health. One student described that despite advancements in education and increased awareness of mental health issues, there is still a significant stigma that exists; however, she believed the nursing students needed to become more comfortable working with mental health patients as “… you are going to have these patients no matter what.”

#### 4.3.3. Desire to Know More about and Interest in Psychiatric Nursing

Despite their concerns, students wanted to learn more about and were interested in psychiatric nursing. When asked about their experience on getting more involvement working around PMH-RNs, they desired to have more direct interaction with nurses and to see PMH-RNs in a greater variety of settings. One student said, “They (nurses) sit in with the doctor, with the patients, and kind of talk about what's going on. We have not had the opportunity to attend those meetings yet and I kind of would like to. I think it would be interesting to hear about and observe how those meetings (go)…” Additionally, following the completion of the questions in the focus group guide, the students had multiple questions about the experiences of the facilitator, an experienced PMH-RN who had worked in a variety of settings. Students asked her to explain more of what she did as a psychiatric nurse, what a typical case load was like, and what the typical hours were. Students' motivation to join the field appeared to stem from a desire to see positive change in patients. They believed psychiatric nursing would allow them to care for patients in ways that promote recovery/remission and an overall better quality of life.

## 5. Discussion

The purpose of this study was to understand the perspectives of administrators, PMH-RNs, and students on factors affecting recruitment and retention of acute-care PMH-RNs. Three commonalities across these groups emerged. First, each group desired more interaction between nursing students and PMH-RNs. Administrators identified preceptorships as a method to recruit students into the PMHN specialty. PMH-RNs perceived that students were not familiar enough with the specialty. Students indicated that clinical experiences do not give them enough interactions with PMH-RNs and asked many questions about the roles of PMH-RNs. As commented by nurses and administrators, experienced PMH-RNs are a great asset for the organization, newly hired nurses, and patients. Based on PMH-RNs suggestions, nursing classroom experiences could include presentations by clinical PMH-RNs to demystify the roles and functions of PMH-RNs and encourage students to choose psychiatric nursing as their clinical specialty.

Nurses believed psychiatric nursing rotations should be mandatory for nursing students, but students thought all nursing students receive some kind of experience and knowledge about psychiatric care, even if not directly through a psychiatric rotation. Undergraduate nursing programs have latitude in meeting accreditation standards of professional nursing organizations and state boards of nursing but are limited by the amount of information needed to be presented within the program's credit hours. Although basic psychiatric nursing knowledge and skills are taught based on national standards [42], programs vary in the type and length of psychiatric clinical experiences, resulting in limited opportunities for learning the in-patient psychiatric nursing role in some programs. Thus, more concerted curricula efforts are needed to attract nurses to practice psychiatric nursing. Developing academic-practice partnerships, practice models that integrate best psychiatric nursing practices with clinical simulation, and basic competencies for psychiatric nursing faculty and preceptors have been identified as priorities to improve PMHN education [35]. Mentoring students by PMH-RNs through classroom presentations or through preceptorships are examples of the types of academic-practice partnerships that will give students an opportunity for additional PMHN experiences without straining current content or increasing clinical credit hours.

Second, all groups addressed safety concerns experienced by PMH-RNs with extensive discussion about violent incidents and how such incidents can physically and psychologically affect nurses. Based on our results, violent encounters with patients were not an infrequent occurrence in psychiatric acute-care settings. While there are some safety protocols and follow-up measures being implemented, nurses expressed their desire to see more investment of resources to prevent violent encounters and support nurses after incidents occur.

At the organizational level, the prevalence of workplace violence is significantly higher in psychiatric settings than in other healthcare settings [9]. A national survey of PMHNs found that less than two-thirds of participants reported feeling safe or very safe in their workplace [23]. Similar to our findings, survey participants cited patient acuity, need for administrative support, lack of staff training, and low staff-to-patient ratios as factors contributing to unsafe work settings. In addition, nurses in our focus groups cited support from colleagues, conducting debriefing “huddles” with consideration for nurses' other duties, and providing quality nursing care after a violent patient-care event as protective factors for retention. Experienced nurses serving as mentors were cited as valued, supportive colleagues, so retirement of nurses may limit the availability of experienced nurses who can serve in this role.

Administrators and nurses in our study indicated their environment is not equipped or suitable for the level of care some patients need. Administrators noted long wait-times to transfer patients with more severe illness to other psychiatric facilities, which reflect the national shortage of psychiatric beds [4]. PMH-RNs also indicated a need for more residential facilities and better housing in the community for patients, suggesting that nurses understood that social determinants of health contributed to patients' relapse and need for hospitalization. Relatedly, a shortage of healthcare providers in psychiatric nursing settings contributed to the inability or difficulty handling violent incidents as suggested in the literature [43]. Nevertheless, it is important to better support psychiatric nursing staff as evidence indicates that workplace assault or violence is positively associated with occupational stress and negatively associated with well-being [9].

Third, nurses and students commented on the stigma of caring for patients with mental illness. While there has been an increased awareness of mental illness in the general population [44], stigma is still associated with mental illness in the US. Negative views about mental illness can translate to negative perceptions of the psychiatric nursing workforce. Stigma within nursing education programs and the belief that medical-surgical experience is needed prior to specializing in PMHN further contribute to recruitment barriers [19, 23]. Phoenix suggested that because psychiatric nursing work is largely relationship-based and lacks the profession's visual signifiers (e.g., stethoscopes and scrubs); it can be a disadvantage for recruiting nurses into the specialty [13]. Students said that they cared for patients with mental health needs in medical-surgical settings, so developing experiences for PMH students in primary care that address patients' physical and mental health needs can enhance PMH-RNs' ability to provide integrated care [45] and may increase students' interest in PMHN.

Previous studies found that at the individual level, negative attitudes of psychiatric nursing as a career [46], stress, burnout [47], and lower job satisfaction [48] may explain the high turnover intention in PMH-RNs. Participants in our focus groups also identified these factors. At the organizational level, lack of in-service education and advanced training [34], negative working relationships [33], and procedural fairness concerning performance appraisal [49] were found to be associated with higher turnover intention. In our study, comments from PMH-RNs suggested that lack of training and interpersonal problems among staff were reasons for leaving the in-patient setting. Although PMH-RNs appreciated administrative support from nurse managers, they also indicated additional types of support were needed. Exploration of leadership roles of unit-level nurse managers may help identify those that enhance staff satisfaction as well as patient care. For example, Perkins, Bamgbade, and Bourdeanu found that higher job satisfaction was related to managers who were able to mentor staff, monitor the unit milieu, and direct unit activities by clarifying expectations and problem-solving [50]. While staffing issues, such as 24-hour coverage and weekend work, are similar across in-patient settings, they may add to PMH-RNs' decisions to take noninpatient positions in PMHN or other specialties, especially if other aspects of the clinical setting are negative.

Addressing nursing turnover is critical as it can decrease the quality of care and increase hiring costs for hospitals [51]. The average cost of turnover for a bedside nurse is $52,350 and a 1% decrease in nurse turnover saves a hospital $380,600 per year [22]. With mental health issues on the rise and increasing turnover and dissatisfaction among the current nursing workforce, it is critical to improve the physical and mental well-being of PMH-RNs and increase retention rates. Additionally, it is imperative government agencies and healthcare systems understand the significant contribution of psychiatric nursing in mental health treatment [13] and ways to recruit nurses into this specialty.

Study limitations include the small sample size. Despite extensive outreach efforts to recruit, only PMH-RNs who had left the acute-care setting agreed to participate, so we did not have the perspective of PMH-RNs who currently work in an acute-care facility which may bias results. Inpatient staffing shortages and high levels of COVID-19-related provider stress may have deterred in-patient PMH-RNs from participating. However, some participants had more than 10 years of experience in the in-patient PMH setting. All participants were recruited from urban healthcare facilities or colleges, which limits generalizability. Additionally, the virtual Zoom sessions may have affected information sharing. Nonetheless, identified themes were congruent with other studies and provide insight into the psychiatric nursing experiences of administrators, PMH-RNs, and students. These experiences provide a basis for recommendations to enhance recruitment and retention of PMH-RNs.

## 6. Conclusions

This qualitative study described perspectives about recruitment and retention of PMH-RNs. Interview data from administrators, PMH-RNs, and students indicated that increased interactions between students and PMH-RNs would increase students' interest in psychiatric nursing as well as satisfy PMH-RNs desire to promote their specialization. Workplace violence and stress, exacerbated by understaffing, lack of understanding of the PMH-RN role, and stigma about mental health were cited as negatively affecting both recruitment and retention of PMH-RN in acute-care settings. Recommendations for improving recruitment and retention include strengthening academic-practice partnerships, increasing resources to prevent and address the aftermath of patient-initiated violence, and promoting the public visibility and importance of psychiatric mental health nursing.

Moving forward, it is crucial to consider the insights provided by this study to formulate effective strategies for improving recruitment, retention, and training of PMH-RNs. By implementing targeted interventions, such as nurse residency programs, continuing education initiatives, and efforts to combat stigma surrounding psychiatric nursing, the aim is to create supportive and conducive working environments that encourage the growth and sustainability of the PMHN workforce. These measures are vital for creating supportive environments that foster the growth and sustainability of the PMHN workforce, ultimately ensuring high-quality mental health care for those in need.

## Figures and Tables

**Table 1:** Themes and illustrative quotations.

Group	Theme	Subtheme	Illustrative quotations
Administrators	(1) Recruitment challenges led to a focus on retention	(a) Recruitment was challenging but slightly improving	(i) “We've had three offers out waiting on two, one has accepted so we are now seeing folks coming and wanting to come into behavioral health.”
			(ii) “I'd say about 2 months ago…we started to see a lot of applications had quite a few new hires, 6–8 months prior to that, it was like, okay, is everything in there [application site] right?”
		(b) Focus on retention	(i) “We have another process that if a staff is injured, we follow up with them and several people involved in that and really doing a dive into supporting them through that process, and I think all of that has been helpful for retention.”
			(ii) “Everybody else is doing increasing wages and doing retention bonuses and all those types of things.”
	(2) Ongoing processes and support for nurses	(a) Varied and individualized onboarding process	(i) “…so we make sure that we're talking about more experiences that the orientee needs to have. Are there areas that we need to reinforce, areas that are going well, those type of things so we can keep tailoring it to the needs of the person…”
		(b) Ongoing formal/informal support provided	(i) “So we have a policy on workplace violence, and we put a lot of effort around staff doing incident reports, if they're witnessing it or if it's happening and to come forward, there's an anonymous way to report it.”
			(ii) “We have that stress first aid program, and then we utilize EAP quite a bit…”
	(3) Needed support for nurses and the profession		(i) “…we brought in individuals who were trained in crisis intervention, how to deal with a violent episode to kind of work through those feeling…I think the ability to have a 911 [emergency] support person to come in and triage, that would be great.”
			(ii) “…keep working on getting started in high schools and getting kids interested in mental health.”

Nurses	(1) Experience and challenges of their profession	(a) Benefits of the profession	(i) “It's just very rewarding watching people recover from a very, very dark place and just seeing that personal growth and self-discovery.”
			(ii) “…one person can have an impact on one other person, and if you can be that person and have an impact and totally change their life.”
		(b) Challenges of the field	(i) “I've worked in some places that have been really unsafe where staff are getting seriously injured or patients are getting injured…and I'm not willing to work in a unit or a facility that has those core safety concerns.”
			(ii) “I've definitely seen a lot of conflict between disciplines and roles when it comes to this career field and my first job there was a lot of conflict because our techs were high school diploma or GED techs, and there was challenges with that. And then my second job, all of our techs had to have at least a bachelor's degree in a related field so that improved a lot of the morale and decreased a lot of the conflict.”
	(2) Sources of current support (formal, informal, and self-care activities)		(i) “That's where I learned most of my stuff is from veteran nurses who have been through it, and so venting to them after a situation like this really is helpful to me and to get their support and feedback about what happens.”
			(ii) “I would say I'm having more phone conversations with people to let some of that out, whereas, you know it used to be face to face, but now it's more on the phone.”
	(3) Need for additional support		(i) “We need to find courage from each other. So, if we just had a little more support, I believe the emotional stuff we go through would be beneficial.”
			(ii) “…but also maybe Q&A would be the right word, for people to just talk with other psych nurses, ask the questions they have or definitely like I've already said, I would love to be a preceptor.”

Students	(1) Mental health experience	(a) Previous/current work and personal experience	(i) “I feel like we've gotten to see a good variety of psychiatric diseases and mental health disorders and all of that just on like Med Surg floors and everything.”
			(ii) “I specifically work at [a rehabilitation facility] and so by working there I feel like I get to work a lot not just in behavioral health but also like helping patients with their physical health…”
		(b) Clinical experience	(i) “So we got to observe quite a few mental status exams, a lot of therapeutic communication and trying to de-escalate situation as well as…their daily routine…the nurses were kind of spread thin so were in the way a lot trying to help them…”
	(2) Concerns about the PMH specialty		(i) “I've been hearing it…just [students] thinking it's all violent patients but it's not. I think that's a big thing that worries some people.”
			(ii) “I also said that my biggest concern is safety.”
	(3) Desire to know more about and interest in psychiatric nursing		(i) “They (nurses) sit in with the doctor, with the patients, and kind of talk about what's going on. We haven't had the opportunity to attend those meetings yet and I kind of would like to. I think it would be interesting to hear about and observe how those meetings [go]…”
			(ii) “I really wish we would be able to follow those nurses on the psych unit…on a nursing standpoint, we're not getting anything really, truly productive out of it.”

## Data Availability

The focus group transcript data used to support the findings of this study are available from the corresponding author upon reasonable request.
